# Isolation and characterisation of irinans, androstane-type withanolides from *Physalis peruviana* L.

**DOI:** 10.3762/bjoc.15.196

**Published:** 2019-08-23

**Authors:** Annika Stein, Dave Compera, Bianka Karge, Mark Brönstrup, Jakob Franke

**Affiliations:** 1 Centre of Biomolecular Drug Research, Leibniz University Hannover, Schneiderberg 38, 30167 Hannover, Germany; 2 Helmholtz Centre for Infection Research, Inhoffenstrasse 7, 38124 Braunschweig, Germany

**Keywords:** androstanes, *Physalis peruviana*, steroids, structure elucidation, withanolides

## Abstract

Withanolides are steroidal lactones widespread in Nightshade plants with often potent antiproliferative activities. Additionally, the structural diversity of this compound class holds much potential for the discovery of novel biological activity. Here, we report two newly characterised withanolides, named irinans, from *Physalis peruviana* with highly unusual truncated backbones that resemble mammalian androstane sex hormones. Based on biomimetic chemical reactions, we propose a model that links these compounds to withanolide biosynthesis. Irinans have potent antiproliferative activities, that are however lower than those of 4ß-hydroxywithanolide E. Our work establishes androwithanolides as a new subclass of withanolides.

## Introduction

Traditional medicine has long been a source of inspiration for modern drug research. An important example is *Withania somnifera,* also known as ashwaghanda or Indian ginseng, which has been used in Ayurvedic medicine to treat a large variety of ailments [1]. Extensive studies revealed withanolides, a class of steroidal lactones, to be primarily responsible for the medicinal effects [1–2]. A large range of pharmacological properties has been assigned to withanolides, with antiproliferative activities being the most potent ones [1]. Withanolides have been also discovered in numerous genera other than *Withania*, for example *Datura*, *Dunalis*, *Iochroma*, *Jaborosa*, *Lycium* and *Physalis* [3], resulting in more than 300 known representatives [3]. *Physalis peruviana* is a withanolide producer of particular relevance as it is widely cultivated for its edible berries [4]. So far, several withanolides have been reported from *P. peruviana* and other *Physalis* species, most prominently physalins, perulactones and 4ß-hydroxywithanolide E (**1**) [5–16]. As part of our ongoing programme focussed on the biochemistry of withanolides, our aim was to gain further insights into the withanolide profile of *P. peruviana*. Here we report irinans A (**2**) and B (**3**), two unusual truncated withanolides with androstane backbones. We show that oxidative, but not acidic or basic conditions enable conversion of the putative precursor 4ß-hydroxywithanolide E (**1**) to irinan A (**2**). Based on this intrinsic reactivity we propose a biosynthetic model that will serve as further guidance for elucidating the enzymatic basis of androstane formation in plants in the future.

## Results and Discussion

**Figure 1:**
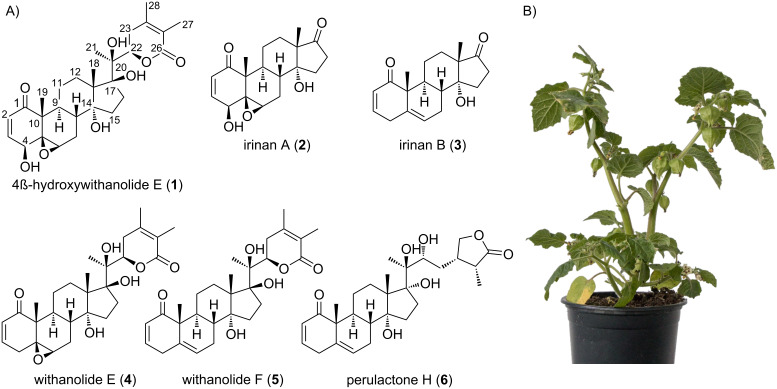
Withanolides from *Physalis peruviana*. A) Structures of the newly characterised truncated withanolides irinan A (**2**) and B (**3**) with an androstane backbone, together with the known withanolides **1** and **4**–**6** isolated from *P. peruviana*. B) *P. peruviana* plant.

To isolate withanolides from *P. peruviana*, we used a purification strategy based on previous reports [17–19]. Nine weeks old whole *P. peruviana* plants (140 g) were extracted with H_2_O/MeOH (3:1) and divided into fractions soluble in petroleum ether, chloroform, and *n*-butanol, respectively. The chloroform fraction was further separated by flash chromatography on a C_18_ stationary phase, resulting in three major subfractions F1–F3. Final purification by preparative HPLC followed by NMR analysis revealed 4β-hydroxywithanolide E (**1**) as the major compound (50 mg) as well as the known metabolites withanolide E (**4**), withanolide F (**5**) and perulactone H (**6**) by comparison to literature data (Figure 1) [9,20].

Two additional compounds attracted our attention based on their unusual ^1^H NMR spectra (Table 1). Both showed two multiplets in the olefinic region, which are highly characteristic for withanolides with A-ring Michael acceptors. However, compared to other withanolides, several signals were missing. Typically, withanolides show five singlets for methyl groups in the aliphatic region, as well as the H-22 oxymethine proton of the lactone moiety. Surprisingly, both compounds showed only two putative methyl signals, and no signal which might correspond to H-22. Thus, we reasoned that both unknown compounds might be truncated withanolide-like compounds.

**Table 1:** ^13^C and ^1^H NMR data (CDCl_3_, 500 MHz, 298 K) of irinans A (**2**) and B (**3**) in comparison to the known compound 4β-hydroxywithanolide E (**1**, CDCl_3_, 400 MHz, 298 K, δ in ppm, *J* in Hz). For carbon numbering see Figure 1 and Figure 2.

	^13^C			^1^H		
**position**	**1**	**2**	**3**	**1**	**2**	**3**

1	201.9	202.0	203.8	–	–	–
2	133.2	132.3	128.1	6.22 (1H, d, 9.9)	6.22 (1H, d, 10.0)	5.91 (1H, ddd, 10.0, 3.1, 1.2)
3	141.4	142.1	145.3	6.92 (1H, dd, 9.9, 6.1)	6.94 (1H, dd, 10.0, 5.8)	6.79 (1H, ddd, 10.0, 5.0, 2.6)
4	70.4	69.9	33.6	3.74 (1H, d, 6.1)	3.79 (1H, dd, 5.8, 2.4)	3.31 (1H, dddddd, 21.3, 2.8, 2.8, 2.8, 2.8, 2.8)^a^ 2.88 (1H, dd, 21.2, 4.9)
5	64.2	63.9	135.7	–	–	–
6	63.1	63.1	124.3	3.28 (1H, br s)	3.37 (1H, m)	5.64 (1H, dt, 5.7, 2.0)
7	26.0	24.9	24.1	2.03 (2H, m)	2.11 (1H, dt, 14.2, 3.1) 1.84 (1H, ddd, 14.1, 11.7, 1.4)	2.08 (1H, m) 1.95 (1H, m)
8	34.3	32.6	35.5	1.83 (1H, m)	1.90 (1H, m)	1.88 (1H, m)
9	36.7	38.1	37.1	1.69 (1H, m)	1.51 (1H, m)	2.10 (1H, m)
10	47.9	47.8	50.9	–	–	–
11	21.5	20.4	21.6	1.72 (1H, m) 1.56 (1H, m)	1.91 (1H, m) 1.46 (1H, m)	2.34 (1H, m) 1.52 (1H, m)
12	29.8	24.3	25.0	2.25 (1H, m) 1.28 (1H, m)	1.66 (1H, d, 13.2) 1.55 (1H, m)	1.86 (1H, m) 1.63 (1H, m)
13	54.6	52.6	52.5	–	–	–
14	81.9	80.9	81.0	–	–	–
15	32.5	30.0	29.9	1.66 (1H, m) 1.59 (1H, m)	1.92 (2H, m)	1.96–1.85 (2H, m)
16	38.0	33.1	33.1	2.72 (1H, m) 1.45 (1H, m)	2.44 (1H, ddd, 18.9, 7.6, 4.1) 2.33 (1H, dt, 18.8, 8.8)	2.35–2.46 (2H, m)
17	87.8	218.0	218.5	–	–	–
18	20.4	17.9	18.1	1.07 (3H, s)	1.01 (3H, s)	1.05 (3H, s)
19	16.9	17.8	19.2	1.42 (3H, s)	1.45 (3H, s)	1.27 (3H, s)
20	79.2	–	–	–	–	–
21	19.8	–	–	1.42 (3H, s)	–	–
22	79.7	–	–	4.88 (1H, dd, 11.8, 5.3)	–	–
23	34.4	–	–	2.51 (2H, m)	–	–
24	150.8	–	–	–	–	–
25	121.6	–	–	–	–	–
26	166.0	–	–	–	–	–
27	12.5	–	–	1.88 (3H, s)	–	–
28	20.8	–	–	1.94 (3H, s)	–	–
14-OH				n.d.	1.41 (1H, br s)	1.41 (1H, br s)
4-OH				n.d.	2.57 (1H, d, 2.50)	-

^a^Apparent dsext. See Figure S19 (Supporting Information File 1) for details. n.d. not detected.

**Figure 2:**
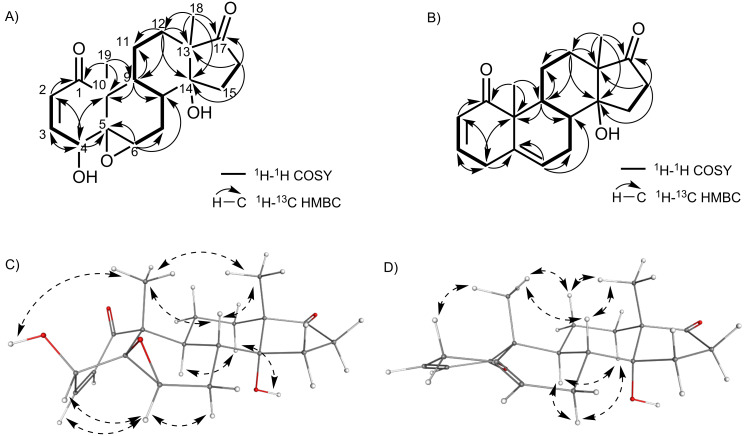
Key NMR correlations. (A) COSY and HMBC correlations for irinan A (**2**). (B) COSY and HMBC correlations for irinan B (**3**). (C) Key NOESY correlations for irinan A (**2**). (D) Key NOESY correlations for irinan B (**3**).

HRESIMS suggested a sum formula of C_19_H_24_O_5_ for the first compound, which was supported by the ^13^C spectrum (Table 1). By comparing the spectrum to NMR data of other withanolides, we quickly identified the Michael system in ring A based on two olefinic protons (δ_H_ 6.94 and 6.22 ppm), a secondary alcohol at C-4 (δ_H_ 3.79 ppm), a 5,6-epoxide (δ_H_ 3.37 (H-6)), and a tertiary alcohol at C-14 (δ_C_ 80.9 ppm). COSY correlations supported by HMBC analysis (Figure 2A) revealed an intact ABCD ring system with a substitution pattern identical to 4β-hydroxywithanolide E (**1**). Only a single, striking difference was noted: C-17 was shifted from 87.8 to 218.0 ppm, strongly suggesting the presence of a ketone instead of an alcohol. In agreement with the predicted sum formula and the absence of all side chain carbons, this completed the structure of the first unknown compound, which we named irinan A (**2**, Figure 1).

The second unknown compound had a sum formula of C_19_H_24_O_3_ based on HRESIMS and ^13^C NMR (Table 1). In contrast to the first compound, no epoxide and no secondary alcohol at C-4 was present, in agreement with the different elemental composition. Instead, ^13^C NMR indicated a double bond at C5–C6 (δ_C_ 135.7 and 124.3 ppm). Otherwise, all spin systems and correlations indicated a typical withanolide ABCD ring system. Again, a carbon with a distinct downfield shift of 218.4 ppm was found, demonstrating the presence of a ketone at C-17. Highly unusually, H-4β appeared as a doublet of sextets (1:5:10:10:5:1) (dsext) in the ^1^H NMR spectrum. This multiplet was explained as “dddddd” by a total of six COSY correlations (Figure S19, Supporting Information File 1). The resulting compound was named irinan B (**3**, Figure 1). A compound of putatively identical structure was isolated from *P. peruviana* before, but only fragmentary physicochemical data has been reported so far [14].

To elucidate the relative stereochemistry of irinans A (**2**) and B (**3**), we analysed NOESY data (Figure 2). In the case of irinan A (**2**), the β configuration of OH-4 was deduced by the NOESY correlation OH-4/CH_3_-19. OH-14 was assigned as α based on the correlations OH-14/H-12α and H-9/H-12α. The 5,6-epoxide was determined as β by a correlation from H-6 to H-3. These assignments are in complete agreement with the relative stereochemistry of 4ß-hydroxywithanolide E (**1**). In irinan B, the configuration of OH-14 could not be unambiguously inferred from NOE data due to the signal overlap of H-15 with H-12 and other protons. As an alternative, OH-14 α configuration was deduced from the chemical shifts of C-12 and C-9, which experience a strong shielding γ-gauche effect for OH-14α configurations [21]. These data indicate a relative stereochemistry of irinan B matching withanolide F (**5**).

**Figure 3:**
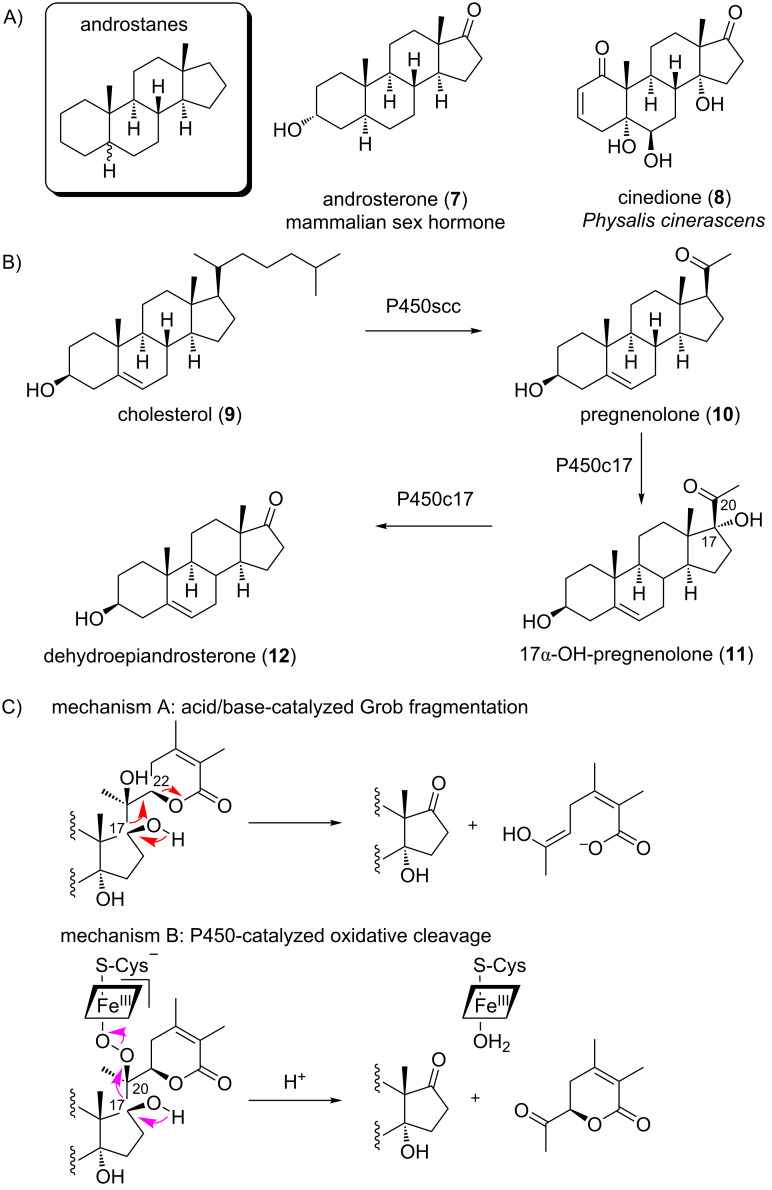
Structures and biosynthesis of androstanes. (A) Androstane backbone and androsterone (**7**) as a typical mammalian sex hormone. Cinedione (**8**) is the only other fully characterised androwithanolide known. (B) Biosynthesis of androstanes in mammals. (C) Possible cleavage mechanisms involved in androwithanolide biosynthesis in plants.

Irinans represent highly unusual withanolide derivatives, as they lack the side-chain lactone ring that is a common structural feature of virtually all known withanolides [3], but possess an androstane backbone instead. While androstanes such as androsterone (**7**) are well-known human sex hormones (Figure 3A) [22], their occurrence in plants is rare [23–26]. Only a single withanolide androstane has been fully characterised before, cinedione (**8**), isolated from *Physalis cinerascens* (Figure 3A) [23]. We propose the name androwithanolides for this withanolide subclass, which so far appears to be characteristic of *Physalis* species.

The biosynthesis of androstanes in mammals requires three enzymatic steps starting from cholesterol (**9**, Figure 3B) [27]. Cholesterol (**9**) is converted to pregnenolone (**10**) by the cytochrome P450 cholesterol side-chain cleavage enzyme (P450scc), which cleaves the C20–C22 bond [27]. Then, the bifunctional P450c17 acts as a 17α-hydroxylase and 17,20-lyase to give rise to androstanes [27]. Related enzymes have not been reported from plants. We searched transcriptome data of *P. peruviana* for putative homologues of these enzymes [28]. The best hits only had amino acid sequence identities of 22–28%, indicating that no P450 enzymes of these clans exist in *P. peruviana*. Although enzymes with similar catalytic activity might have evolved convergently in plants, the different substitution pattern in the side chain suggests that a side-chain cleavage mechanism distinct from mammals is involved. While the order of oxidative steps in withanolide biosynthesis is still completely elusive [29], we propose that this fragmentation occurs at a late stage, when most typical withanolide functionalisations have already been introduced. Indeed, irinan A (**2**), irinan B (**3**) and cinedione (**8**) can be directly linked to the known withanolides 4ß-hydroxywithanolide E (**1**), withanolide F (**5**) and withanolide S [23], respectively (Figure S20, Supporting Information File 1). If the fragmentation occurred early in the biosynthesis, this would imply that several biosynthetic enzymes have to tolerate substrates without the lactone side chain. We therefore propose that the side-chain cleavage enzyme in withanolide biosynthesis acts at a late stage, using common pathway end products such as 4ß-hydroxywithanolide E (**1**) as its substrates. Two mechanisms are conceivable for this transformation (Figure 3C): A non-oxidative Grob fragmentation could make use of a push–pull mechanism between C-17 and C-22, building on acid–base catalysis. Alternatively, an enzyme could cleave the C17–C20 diol oxidatively. Several P450 enzymes have been reported to be capable of cleaving diols, presumably via a ferric peroxo intermediate (Figure 3C) [30–31].

**Figure 4:**
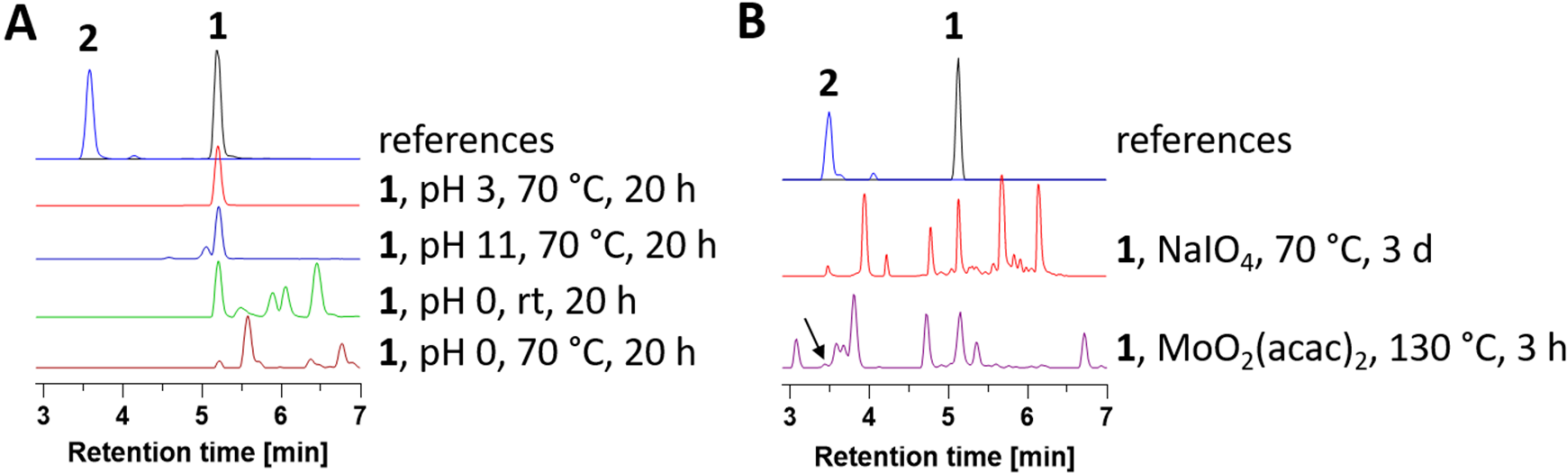
Intrinsic reactivity of 4ß-hydroxywithanolide E (**1**) under acidic/basic and oxidative conditions, respectively. (A) LC–MS chromatograms (ELS detection) of **1** incubated at different pH values. (B) LC–MS chromatograms (UV detection at 200–400 nm) of **1** treated with different oxidative reagents. The formation of **2** in the NaIO_4_ reaction was confirmed by NMR analysis. See also Figure S21 in Supporting Information File 1 for extracted ion chromatograms (EICs).

To gain further insights into the biosynthetic route and to exclude that androwithanolides are isolation artefacts [32], we exposed 4ß-hydroxywithanolide E (**1**) as the likely precursor to irinan A (**2**) to various chemical conditions (Figure 4). In general, **1** was stable in all solvents tested, namely chloroform, methanol, DMSO and acetonitrile (data not shown). Treatment with acid at pH 3 caused no reaction at all when heating up to 70 °C (Figure 4A). At pH 0, several unidentified compounds appeared, but not irinan A (**2**). In basic conditions, only a single unidentified product was formed at pH 11 and 70 °C. Next, we tested whether **1** could be oxidatively cleaved [33]. Incubation of **1** with NaIO_4_ at room temperature did not result in any reaction (data not shown). However, although it has been reported that periodates are not capable of cleaving ditertiary glycols [33–34], we noted formation of small quantities of irinan A (**2**) when performing the reaction at 70 °C (Figure 4B and Figure S21 in Supporting Information File 1). The identity of irinan A (**2**) was verified by isolation of the corresponding compound by preparative HPLC (4% yield) followed by NMR analysis. This result confirms our NMR-based stereochemical assignment and unambiguously links irinan A (**2**) to 4ß-hydroxywithanolide E (**1**). We also performed an oxidative cleavage reaction with catalytic amounts of MoO_2_(acac)_2_ in DMSO as described by García et al. [34], which also led to the formation of trace amounts of irinan A (**2**). Our experiments suggest that irinan A (**2**) and most likely all androwithanolides are not isolation artefacts but true natural products, which require an oxidative enzyme to facilitate the C–C bond cleavage. Future studies will shed light on the enzymatic basis of androwithanolide formation.

Considering the potent bioactivities of androstanes as well as withanolides, we wondered whether the loss of the side-chain lactone would negatively impact the antiproliferative activity. Irinan A (**2**) and B (**3**) together with 4ß-hydroxywithanolide E (**1**) as a positive control were evaluated against a panel of four cell lines (Table 2). In our assays we observed decreasing activities during the third and fourth replicates, resulting in large standard deviations and potentially indicating limited stability of these compounds. Nonetheless, EC_50_ values of 4ß-hydroxywithanolide E (**1**) were in good agreement with previously published values [14,35–36]. Irinans A (**2**) and B (**3**) were 1.3 to 10-fold less active than 4ß-hydroxywithanolide E (**1**), with the exception of irinan B (**3**) in A549 cells, which was equipotent. However, irinan A (**2**) and B (**3**) samples had a purity of 90% and 80%, respectively. We therefore cannot exclude that unidentified impurities, which could not be removed by repeated preparative HPLC, obscure the true EC_50_ values of irinans. We conclude that irinans possess potent antiproliferative activity, that is however reduced compared to 4ß-hydroxywithanolide E (**1**). Our results demonstrate the importance of the lactone side chain for bioactivity.

**Table 2:** Antiproliferative activities in different cell lines. Data indicate EC_50_ values ± SD in µM. A549 = human lung carcinoma; L929 = mouse fibroblast; KB-3-1 = human cervix carcinoma; MCF-7 = human breast cancer cell line.

Compound	A549	L929	KB-3-1	MCF-7

4ß-hydroxywithanolide E (**1**)	3.74 ± 0.50	0.27 ± 0.30	1.11 ± 0.98	10.65 ± 6.18
irinan A (**2**)^a^	5.01 ± 5.27	2.29 ± 0.88	4.62 ± 5.76	17.88 ± 7.27
irinan B (**3**)^b^	3.45 ± 1.91	1.68 ± 1.78	2.40 ± 2.32	13.56 ± 9.18
staurosporine (positive control)	1.19 ± 0.99	<0.003	0.04 ± 0.01	0.16 ± 0.02
auranofin (positive control)	>7.03	2.35 ± 0.83	1.59 ± 0.37	2.06 ± 0.60

^a^Estimated 90% purity based on ^1^H NMR. ^b^Estimated 80% purity based on ^1^H NMR.

## Conclusion

We have discovered and characterised irinans A and B, two new withanolides from *P. peruviana* with truncated backbones. They resemble mammalian sex hormones of the androstane class. The relative stereochemistry was elucidated based on NOESY analysis. Chemical studies support a model that these compounds are formed by an oxidative process. We propose the name androwithanolides for this withanolide subclass.

## Experimental

### General experimental procedures

Seeds of *Physalis peruviana* were obtained from FloraSelf, Sperli and Quedlinburger Saatgut. Plants were initially grown in seed starter soil (Kölle’s Beste Anzuchterde) and later transferred to potting soil (Kölle’s Beste Pflanzerde). Plants were grown under LED illumination (SANlight S2W) at 350 µmol s^−1^ m^−2^ PPFD with a 12 h photoperiod and at 18–25 °C without temperature and humidity control. Plants were watered twice per week with tap water as needed.

NMR spectra were recorded using Bruker Ascend^TM^ 400 or DRX 500 MHz spectrometers operating at 400 and 500 MHz for ^1^H NMR and at 100 and 125 MHz for ^13^C NMR where CDCl_3_ was used as solvent. Chemical shifts were referenced relative to the residual solvent signal of CDCl_3_ (δ_H_ = 7.26 ppm, δ_C_ = 77.16 ppm) and expressed in δ values (ppm), with coupling constants reported in Hz. Analysis was conducted with TopSpin (Version 4.0.6, Bruker). ATR-IR analysis was performed for the range of 400–4000 cm^−1^ using a Shimadzu IRAffinity 1S spectrometer with samples dissolved in chloroform. Optical rotations were measured with a Perkin Elmer 341 polarimeter. Using methanol as solvent, the wavelength for maximum absorption was determined on a Jasco V-630 spectrophotometer. Flash purification was performed on a Biotage Isolera One using columns described below. HRMS measurements were carried out on a Waters Alliance 2695 HPLC coupled to a Micromass LCT Premier mass spectrometer.

For analytical and preparative LC–MS a Waters instrument was used consisting of a Waters 2767 autosampler, Waters 2545 pump system, Waters 2998 diode array detector, Waters 2424 ELS detector, and a Waters SQ Detector 2 for mass spectrometry in ESI^+^ and ESI^–^ modes between *m*/*z* 150 and 1000. In analytical mode, a Phenomenex Kinetex column (2.6 µm, C_18_, 100 Å, 4.6 × 100 mm) was used with a gradient of [solvent A: H_2_O + 0.05% formic acid; solvent B: acetonitrile + 0.045% formic acid; gradient: 10% to 90% B over 10 min, 1 mL/min]. Samples were dissolved to a concentration of 10 mg/mL in MeOH and 20 µL injected. In preparative mode, a Phenomenex Kinetex Axia column (5 µm, C_18_, 100 Å, 21.2 × 250 mm) equipped with a Phenomenex Security Guard precolumn (Luna, C_5_, 300 Å) was used in combination with the separation gradient described below.

### Extraction and isolation of withanolides

140 g of 9 weeks old, whole *Physalis peruviana* plants were frozen in liquid nitrogen and ground to a fine powder. The powder was extracted with 500 mL H_2_O/MeOH (3:1) at room temperature for 3 h. After filtration and evaporation of the solvent under reduced pressure, the crude extract was resuspended in 300 mL H_2_O and defatted with 300 mL petroleum ether. The remaining aqueous layer was further extracted with 2 × 300 mL CHCl_3_ followed by 2 × 300 mL *n*-BuOH. This resulted in a 660 mg petroleum ether fraction, 386 mg CHCl_3_ fraction and 1174 mg *n*-BuOH fraction.

The CHCl_3_ fraction was separated via reversed-phase flash chromatography (Biotage SNAP KP-C18-HS 30 g column) with a H_2_O/MeOH gradient. Samples were adsorbed onto Celite under reduced pressure for dry loading. A gradient from 30% to 95% MeOH was used. Fractions were pooled guided by UV maximum absorbance to form main fraction F1 (subfractions 1–21, 102 mg), F2 (subfractions 22–31, 12 mg) and F3 (subfractions 32–45, 48 mg). No withanolides were detected in F2 based on LC–MS analysis and therefore discarded.

Fraction F1 was further separated by preparative LC–MS. The sample was dissolved in MeOH to a concentration of 15 mg/mL. 100 µL was injected per run. A separation gradient was used [solvent A: H_2_O + 0.05% formic acid; solvent B: acetonitrile + 0.045% formic acid; gradient: 10% to 90% B over 10 min, 20 mL/min]. The post-column flow was split (100:1) and the minority flow made up to 1 mL/min with MeOH + 0.045% formic acid for in-line analysis by UV, ELSD and MS. The majority flow was collected. The following peaks were collected and identified by NMR: *t*_R_ = 5.8–6.0 min (irinan A (**2**), 6 mg); 7.2–8.0 min (4β-hydroxywithanolide E (**1**), 49 mg); 9.0–9.2 min (irinan B (**3**), 1 mg). The collected fractions were evaporated under reduced pressure using a Christ RVC 2-25 CDplus rotational vacuum concentrator.

Main fraction F3 was also separated by preparative LC–MS as described above, yielding the known compounds withanolide E (**4**) (*t*_R_ = 7.3–7.6 min, 6 mg), perulactone H (**6**) (*t*_R_ = 7.6–7.8 min, 9 mg) and withanolide F (**5**) (*t*_R_ = 7.8–8.5 min, 9 mg) which were identified by NMR [20].

### Analytical data

4β-Hydroxywithanolide E (**1**) was isolated as a white crystalline powder. NMR data of **1** is listed in Table 1. All spectroscopic properties matched literature data [20].

Irinan A (**2**): white crystalline powder; [α]_D_^20^ +10.48 (β = 0.62; MeOH); UV (MeOH) λ_max_ (log ε) 239 nm (3.93); IR (ATR, CHCl_3_) ν_max_: 3460, 2967, 2930, 1734, 1674, 1454, 1373, 1092, 1036, 986, 922, 754 cm^−1^; for ^1^H and ^13^C data see Table 1; HRESIMS *m*/*z*: [M + Na]^+^ calcd for C_19_H_24_O_5_Na^+^, 355.1516; found, 355.1519.

Irinan B (**3**): white crystalline powder; [α]_D_^20^ –10.00 (β = 0.06; MeOH); UV (MeOH) λ_max_ (log ε) 251 nm (3.94); IR (ATR, CHCl_3_) ν_max_: 3402, 2955, 2930, 1682, 1383, 1259, 1215, 1136, 1088, 1016, 966, 806, 748 cm^−1^; for ^1^H and ^13^C data see Table 1; HRESIMS *m*/*z*: [M + Na]^+^ calcd for C_19_H_24_O_3_Na^+^, 323.1618; found, 323.1626

### BLAST search of known androstane biosynthesis enzymes

The known androstane biosynthesis enzymes *Homo sapiens* P450scc (UniProtKB accession P05108) and *Homo sapiens* P450c17 (P05093) were used to search reported *Physalis peruviana* transcriptome data [28] via the tBLASTn algorithm. Both enzymes yielded several full-length hits with amino acid sequence identities of 22–28%.

### Oxidative cleavage of 4β-hydroxywithanolide E (**1**) to irinan A (**2**) by NaIO_4_

57.8 mg of NaIO_4_ (270.2 µmol, 7.0 equiv) in 400 µL hot H_2_O was added to 19.4 mg 4ß-hydroxywithanolide E (**1**, 38.6 µmol, 1.0 equiv) in 1 mL MeOH. The reaction was incubated at 70 °C for 72 h in a heat block with shaking at 1000 rpm. After that time a peak with *m*/*z* 315 corresponding to [M + H − H_2_O]^+^ with a retention time of 3.5 min was observed by LC–MS, co-eluting with authentic irinan A (**2**). The reaction mixture was separated by preparative LC–MS as described above to give a white crystalline powder (0.5 mg, 4%), which was confirmed to be irinan A (**2**) by ^1^H NMR spectroscopy.

### Oxidative cleavage of 4β-hydroxywithanolide E (**1**) to irinan A (**2**) by MoO_2_(acac)_2_

2 µL of a MoO_2_(acac)_2_ stock solution in DMSO (100 µg/µL, 0.6 µmol, 0.02 equiv) was added to 14.6 mg hydroxywithanolide E (**1**, 29.0 µmol, 1.0 equiv) in 100 µL DMSO. The reaction was incubated at 130 °C for 3 h in an oil bath with stirring at 400 rpm. After that time a peak with *m*/*z* 315 corresponding to [M + H − H_2_O]^+^ with a retention time of 3.5 min was observed, co-eluting with authentic irinan A (**2**). H_2_O (5 mL) and CHCl_3_ (5 mL) were added to the reaction mixture. The layers were separated and the aqueous phase was extracted with chloroform (3 × 5 mL). Combined organic layers were washed with water (5 mL), dried over MgSO_4_, filtered and the solvent was evaporated under reduced pressure. The resulting crude reaction product was then analysed by analytical HPLC as described above.

### Antiproliferative assays

The effect of compounds on cell viability was probed with a WST-1 test using the procedure of Ishiyama et al. [37] as modified by Sasse et al. [38]. The following cell lines were used: mouse fibroblast cell line L929 (DSM ACC 2), human cervix carcinoma cell line KB-3-1 (DSM ACC 158), the human lung carcinoma cell line A549 (DSMZ ACC 107) and human breast cancer cell line MCF-7 (DSM ACC 115). The subconfluent cells were briefly washed with Earle’s Balanced Salt Solution (Gibco) without Ca and Mg, trypsinized and re-suspended in Dulbecco’s modified eagle’s medium that contained 5% fetal bovine serum (FBS; L929, KB-3-1, A549) or Roswell Park Memorial Institute medium that contained 5% FBS, 0.5% Minimum Essential Medium Non-Essential Amino Acids, Gibco (MEM NEAA), 0.5% GlutaMAX (Gibco) and insulin at 5 μg/mL (MCF-7). 25 µL of serial dilutions of the test compounds (64–0.06 µg/mL, that were made with a pipetting robot (epMotion, Eppendorf, Hamburg, Germany), were added to 25 μL aliquots of a cell suspension (1500 cells for KB-3-1, L929 and A549, 3000 cells for MCF-7) in 384 well microtiter plates. Blank and solvent controls were incubated under identical conditions. After an incubation period of 5 days, 3 μL WST-1 (ready to use solution by Roche) was added. The incubation time of the plates at 37 °C varied between the cell lines from 20 min for KB-3-1 and A549, L929 for 30 min, and 2 h for MCF-7 before measuring absorbance at 450 nm (reference 600 nm) with an Infinite 200 PRO plate reader (Tecan, Männedorf, Switzerland). As positive control compounds, Auranofin and Staurosporin were applied. The absorbance of the solvent control was set to 100%. The EC_50_ values were determined with Sigma Plot. All data are average values from four biological replicates.

## Supporting Information

File 1 NMR, MS, UV and IR spectra of irinan A (**2**) and irinan B (**3**). NMR data of withanolide E (**4**), withanolide F (**5**) and perulactone H (**6**).
