# Supplementary material for: Isolation and characterisation of irinans, androstane-type withanolides from Physalis peruviana L
Source: Beilstein J Org Chem. 2019 Aug 23;15:2003–12. doi: 10.3762/bjoc.15.196 (PMC6720484; doi:10.3762/bjoc.15.196)
Supplement: File 1 — NMR, MS, UV and IR spectra of irinan A (2) and irinan B (3). NMR data of withanolide E (4), withanolide F (5) and perulactone H (6). [file Beilstein_J_Org_Chem-15-2003-s001.pdf]

BEILSTEIN JOURNAL OF ORGANIC CHEMISTRY

Supporting Information

for

Isolation and characterisation of irinans, androstane-type withanolides from *Physalis peruviana* L.

Annika Stein, Dave Compera, Bianka Karge, Mark Brönstrup and Jakob Franke

Beilstein J. Org. Chem. **2019,** *15,* 2003–2012. doi:10.3762/bjoc.15.196

NMR, MS, UV and IR spectra of irinan A (2) and irinan B (3). NMR data of withanolide E (4), withanolide F (5) and perulactone H (6)

License and Terms: This is a supporting information file under the terms of the Creative Commons Attribution License (http://creativecommons.org/licenses/by/4.0). Please note that the reuse, redistribution and reproduction in particular requires that the authors and source are credited.
The license is subject to the *Beilstein Journal of Organic Chemistry* terms and conditions: (https://www.beilstein-journals.org/bjoc)

Figure S1. HRESIMS spectrum of **2**

Figure S2. ^{1}H NMR spectrum of **2** in $CDCl_3$ at 298.0 K (500 MHz)

Figure S3. ^{13}C NMR spectrum of **2** in $CDCl_3$ at 298.0 K (125 MHz)

Figure S4. ^{1}H,^{1}H COSY spectrum of **2** in $CDCl_3$ at 298.0 K (500 MHz)

Figure S5. HSQC spectrum of **2** in $CDCl_3$ at 298.0 K (500 MHz; 125 MHz)

Figure S6. HMBC spectrum of **2** in $CDCl_3$ at 298.0 K (500 MHz; 125 MHz)

Figure S7. NOESY spectrum of **2** in $CDCl_3$ at 298.0 K (500 MHz)

Figure S8. IR spectrum of **2**

Figure S9. UV–vis spectrum of **2** in MeOH

Figure S10. HRESIMS spectrum of **3**

Figure S11. ^{1}H NMR spectrum of **3** in $CDCl_3$ at 298.0 K (500 MHz)

Figure S12. ^{13}C NMR spectrum of **3** in $CDCl_3$ at 298.0 K (125 MHz)

Figure S13. ^{1}H,^{1}H COSY spectrum of **3** in $CDCl_3$ at 298.0 K (500 MHz)

Figure S14. HSQC spectrum of **3** in $CDCl_3$ at 298.0 K (500 MHz; 125 MHz)

Figure S15. HMBC spectrum of **3** in $CDCl_3$ at 298.0 K (500 MHz; 125 MHz)

Figure S16. NOESY spectrum of **3** in $CDCl_3$ at 298.0 K (500 MHz)

Figure S17. IR spectrum of **3**

Figure S18. UV–vis spectrum of **3** in MeOH

Figure S19. Origin of an unusual dddddd multiplet.

Figure S20. Proposed biosynthetic precursors of irinan A (**2**), B (**3**) and the previously reported androwithanolide cinedione (**8**).

Figure S21. Intrinsic reactivity of 4ß-hydroxywithanolide E (**1**) in oxidative conditions.

Table S1. ^{1}H NMR data ($CDCl_3$, 400 MHz, 298 K) of withanolide E (**4**), withanolide F (**5**) and perulactone H (**6**) in comparison with literature data (δ in ppm, *J* in Hz).

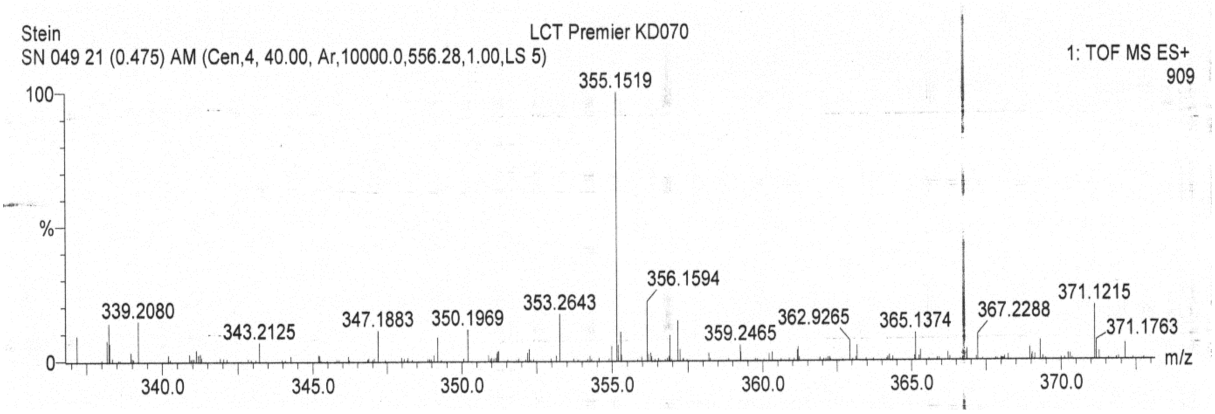

Figure S1. HRESIMS spectrum of **2**.

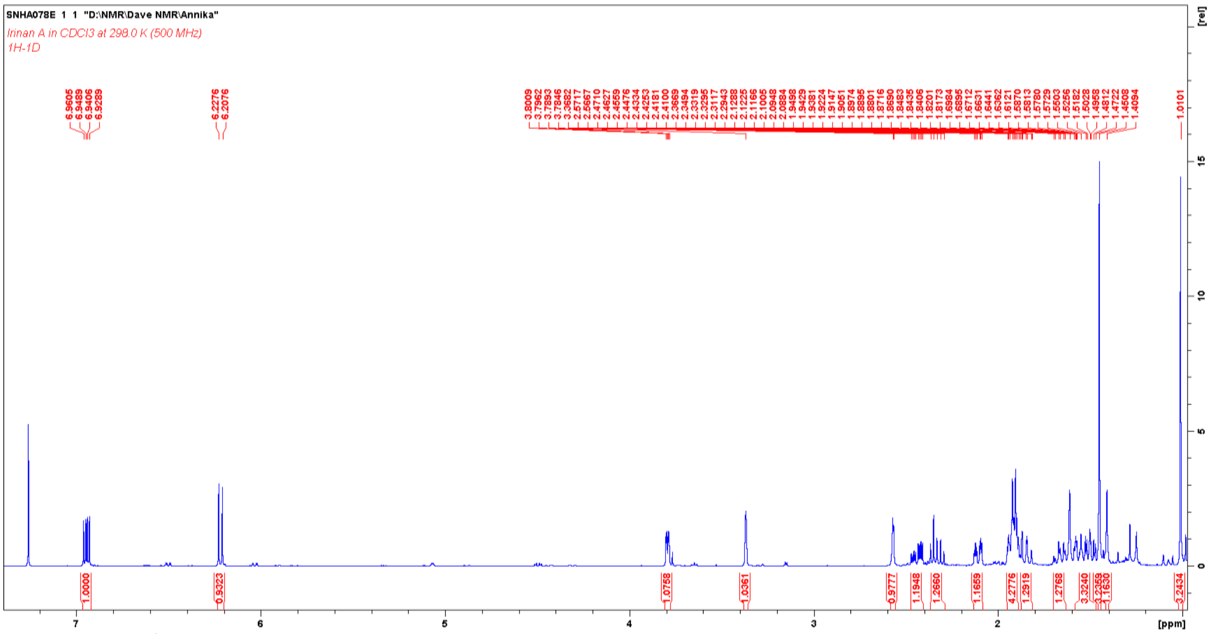

Figure S2. ^{1}H NMR spectrum of **2** in $CDCl_3$ at 298.0 K (500 MHz).

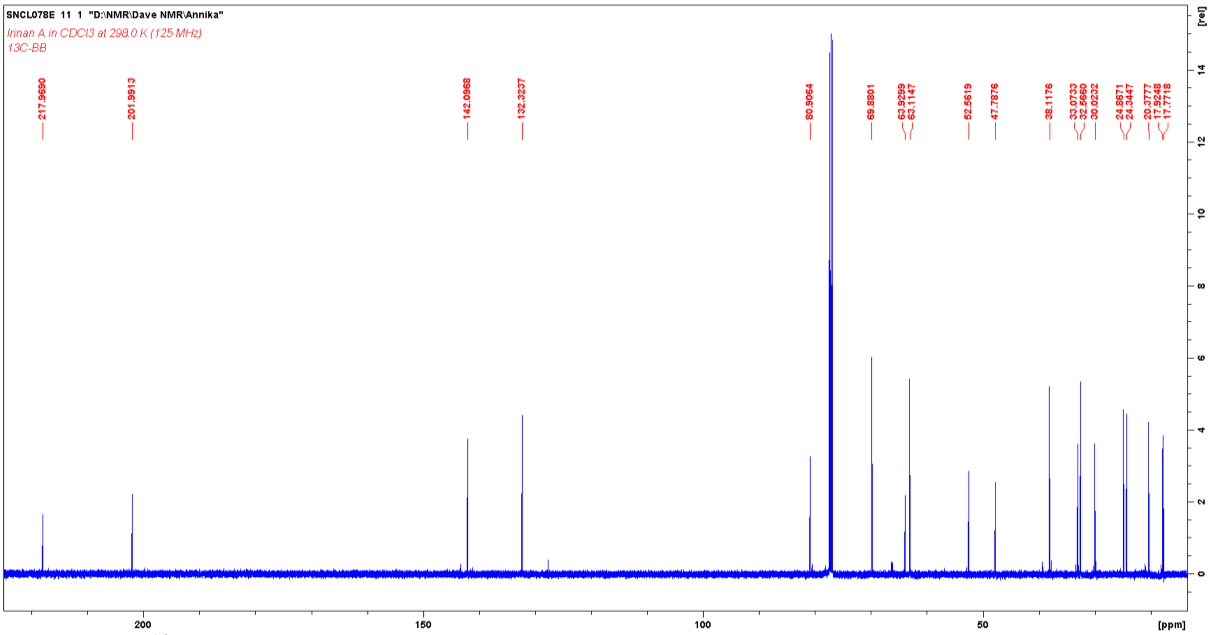

Figure S3. ^{13}C NMR spectrum of **2** in $CDCl_3$ at 298.0 K (125 MHz).

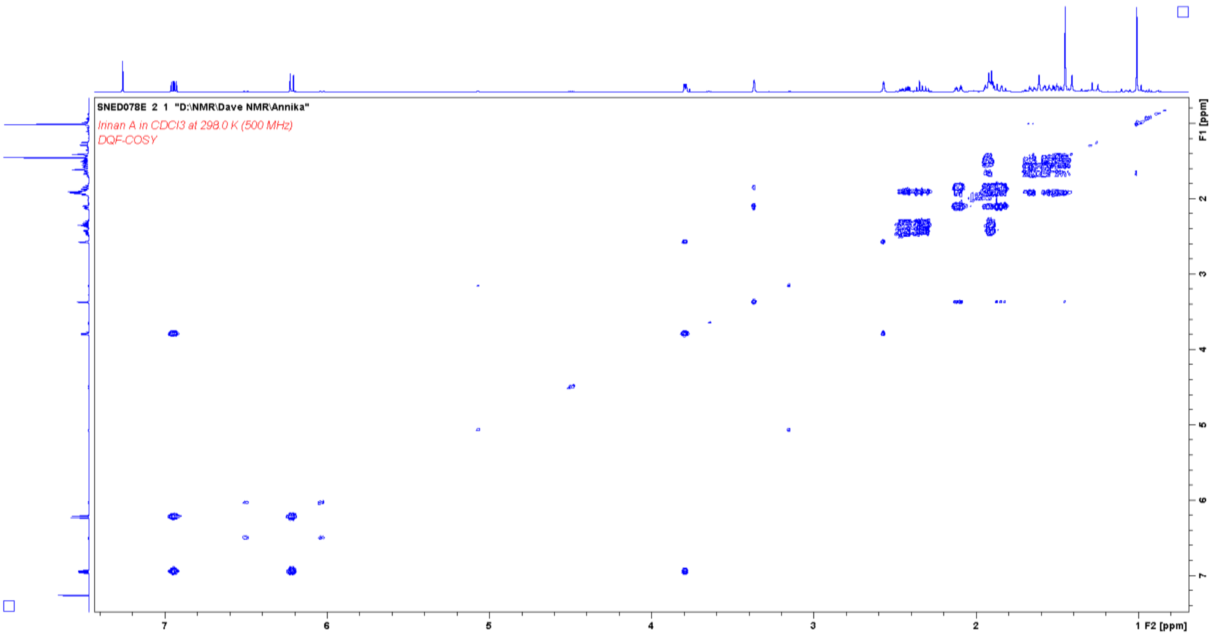

Figure S4. $^{1}H,^{1}H$ COSY spectrum of **2** in $CDCl_3$ at 298.0 K (500 MHz).

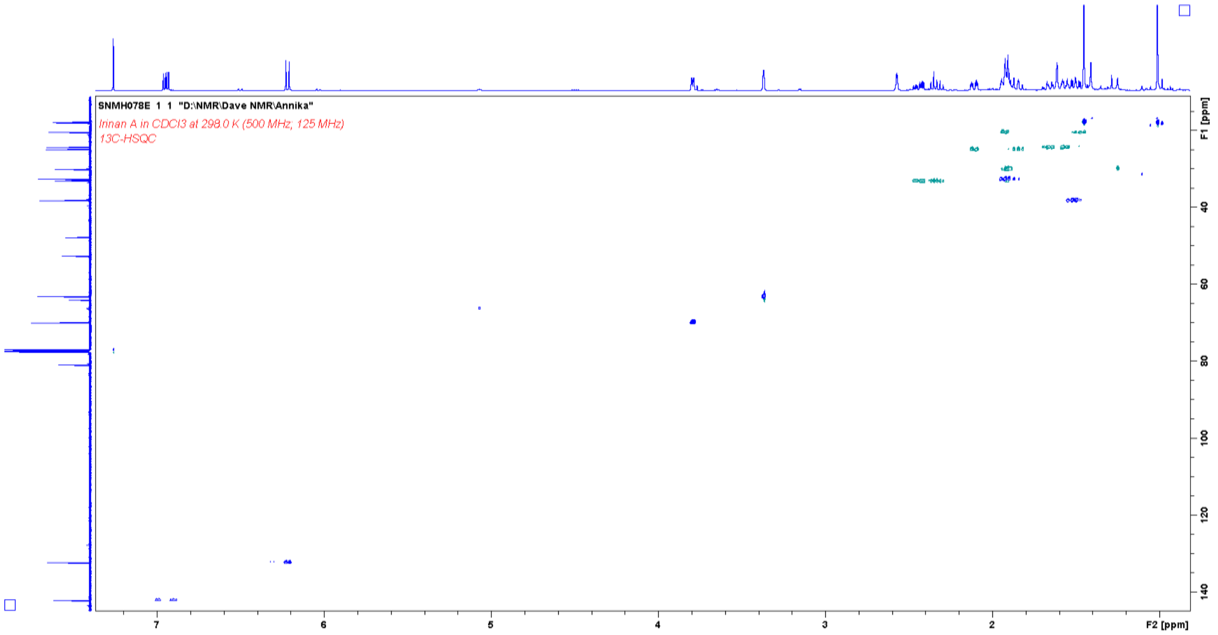

Figure S5. HSQC spectrum of **2** in $CDCl_3$ at 298.0 K (500 MHz; 125 MHz).

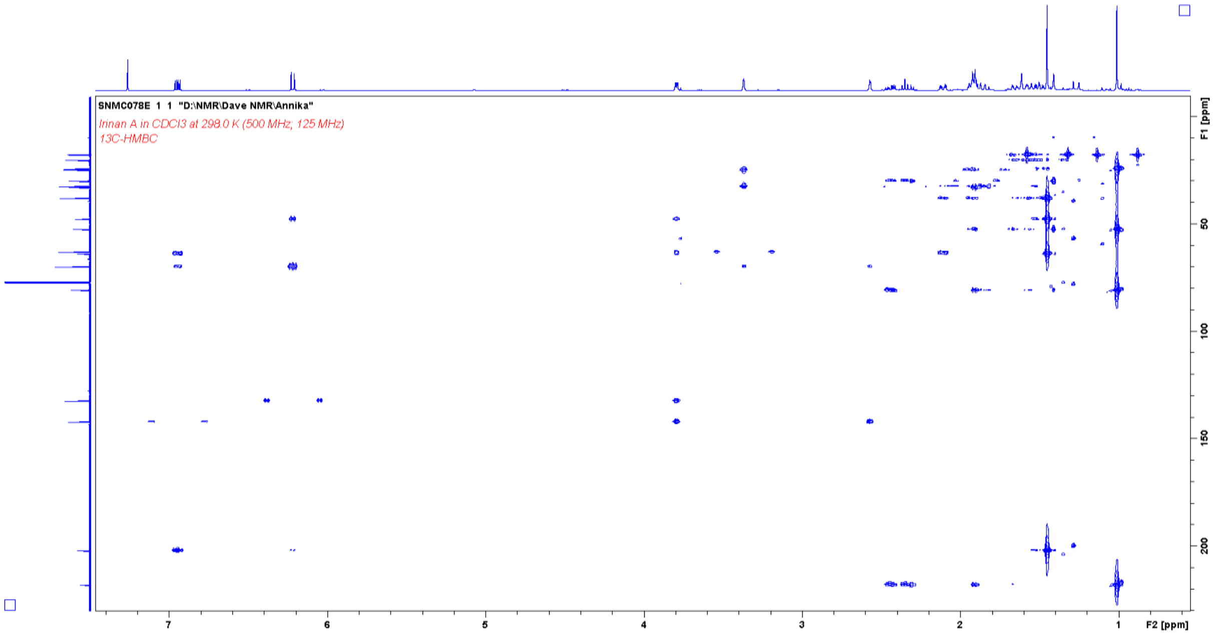

Figure S6. HMBC spectrum of **2** in $CDCl_3$ at 298.0 K (500 MHz; 125 MHz).

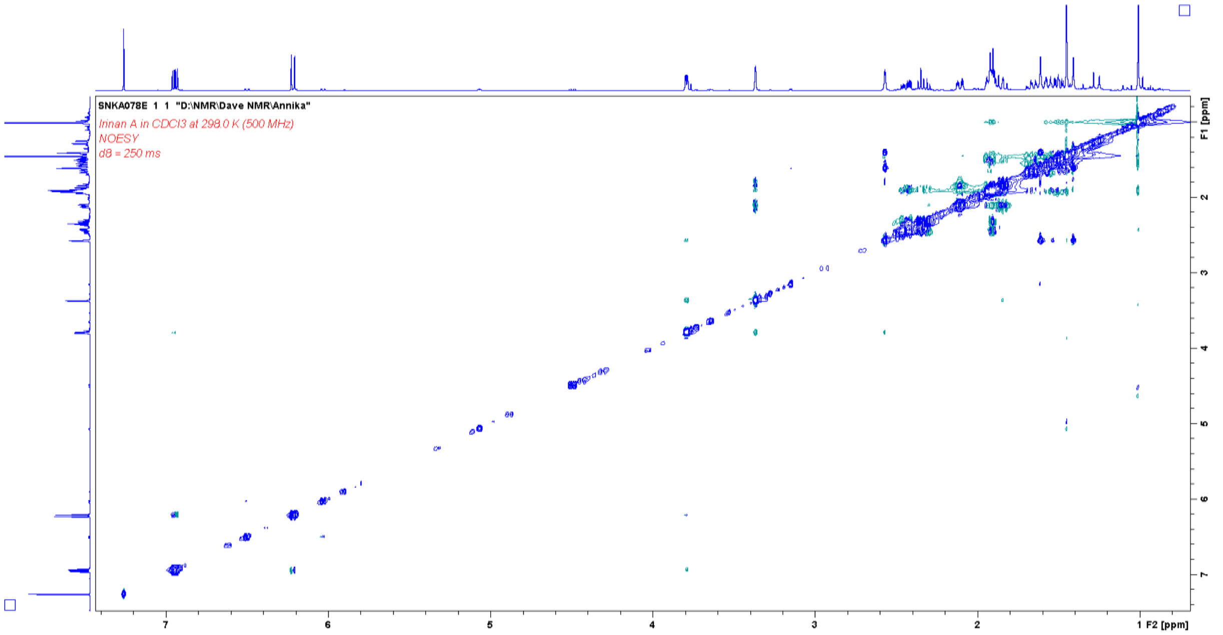

Figure S7. NOESY spectrum of **2** in $CDCl_3$ at 298.0 K (500 MHz).

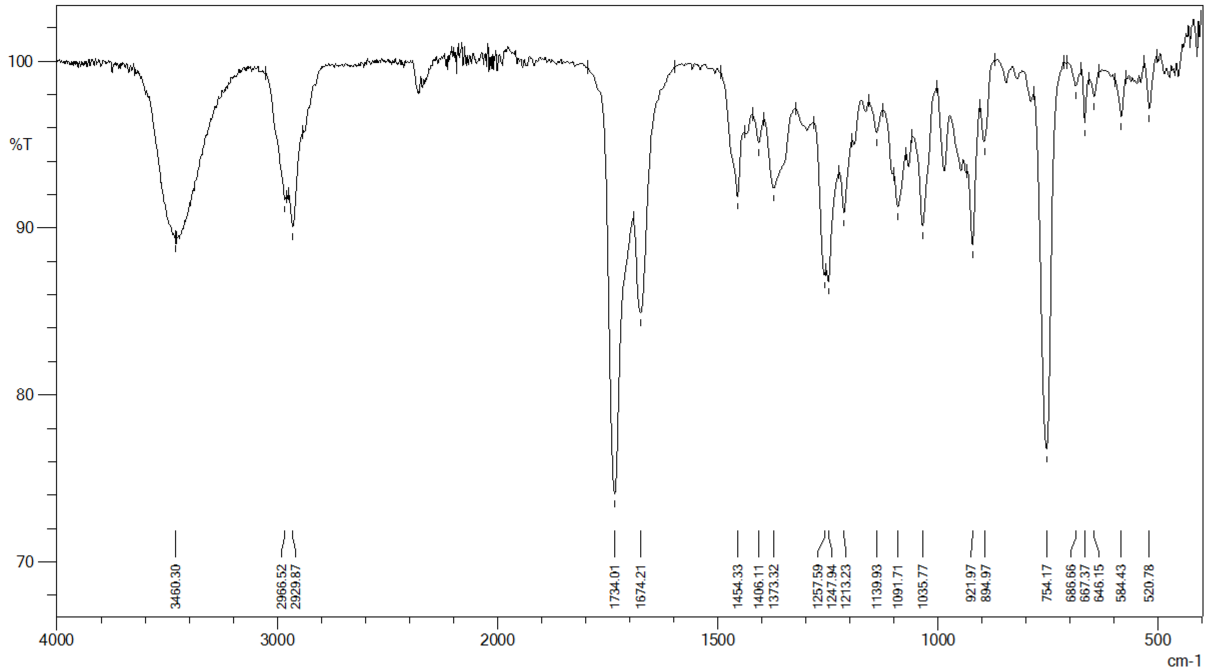

Figure S8. IR spectrum of **2.**

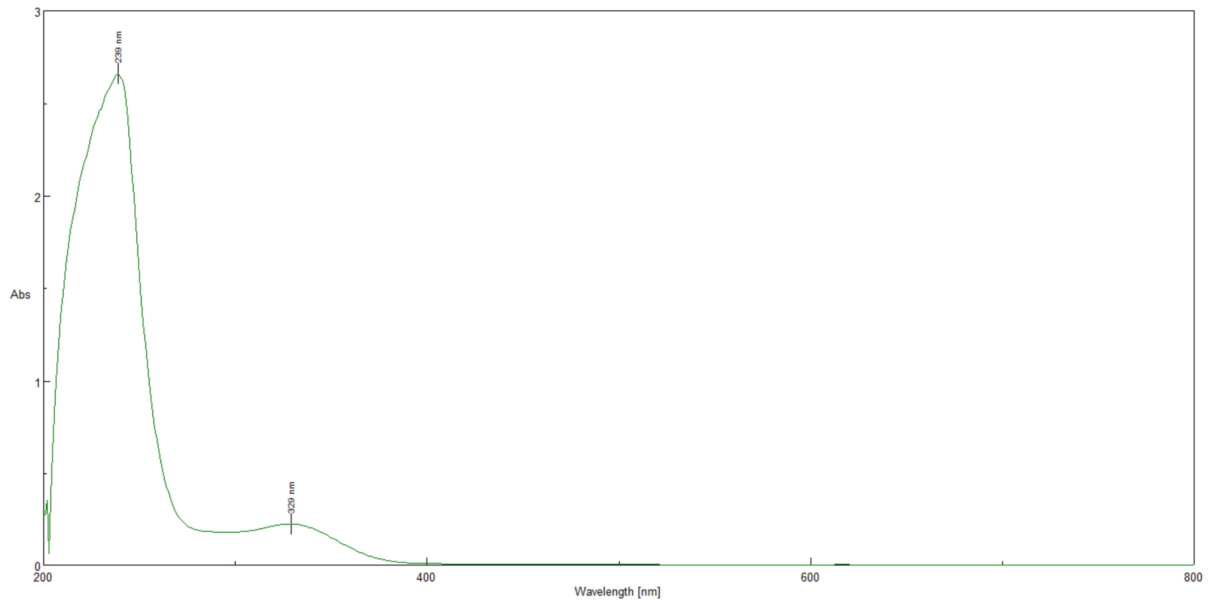

Figure S9. UV–vis spectrum of **2** in MeOH.

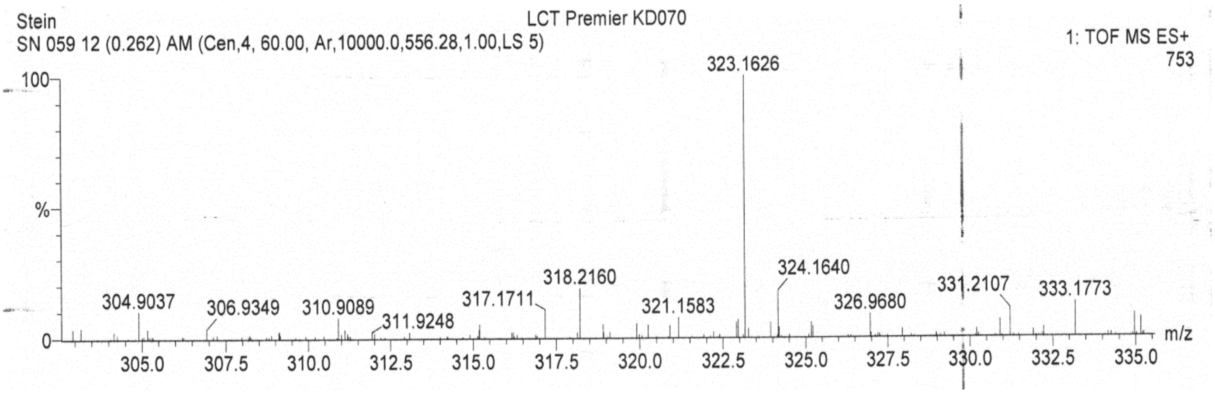

Figure S10. HRESIMS spectrum of **3**.

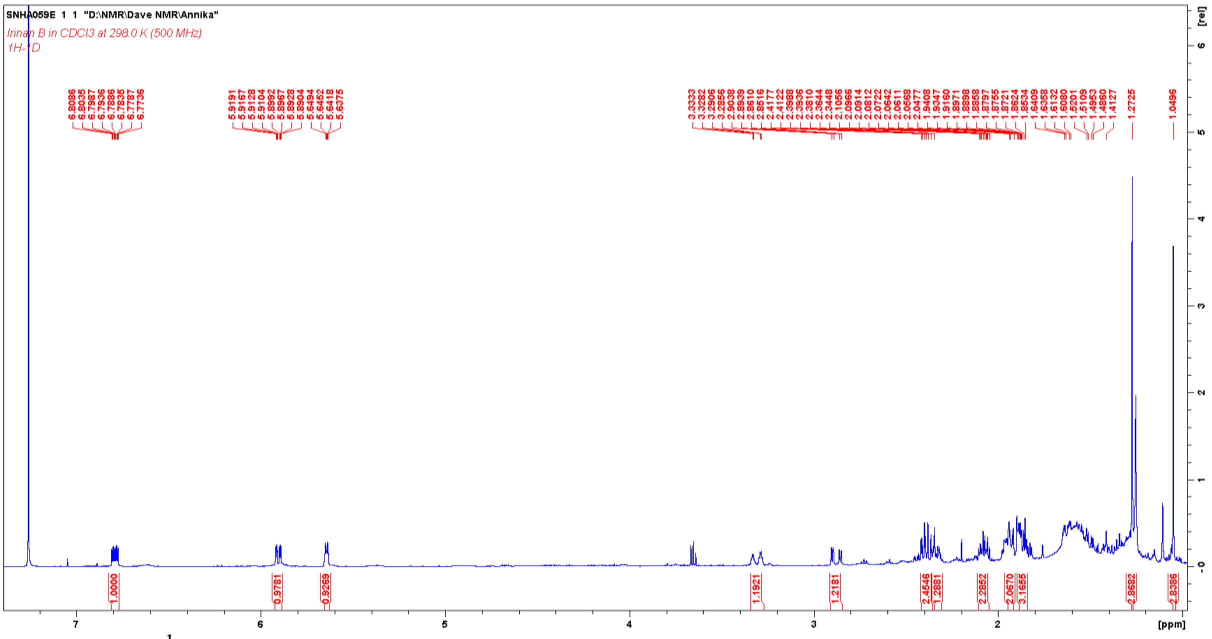

Figure S11. ^{1}H NMR spectrum of **3** in $CDCl_3$ at 298.0 K (500 MHz).

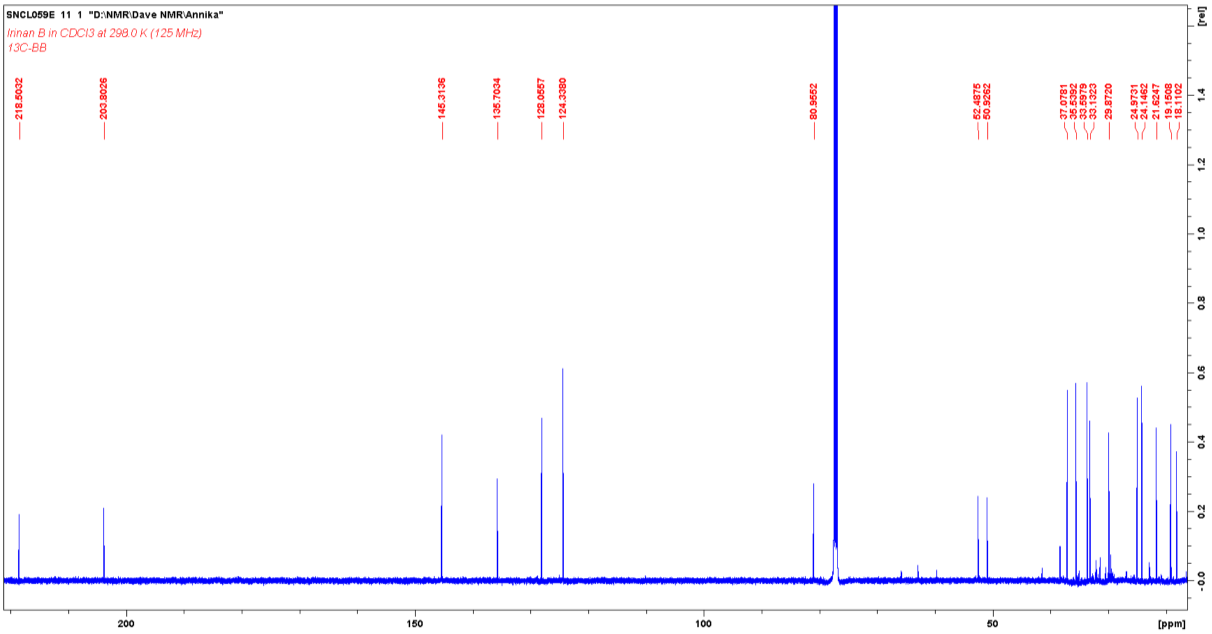

Figure S12. ^{13}C NMR spectrum of **3** in $CDCl_3$ at 298.0 K (125 MHz).

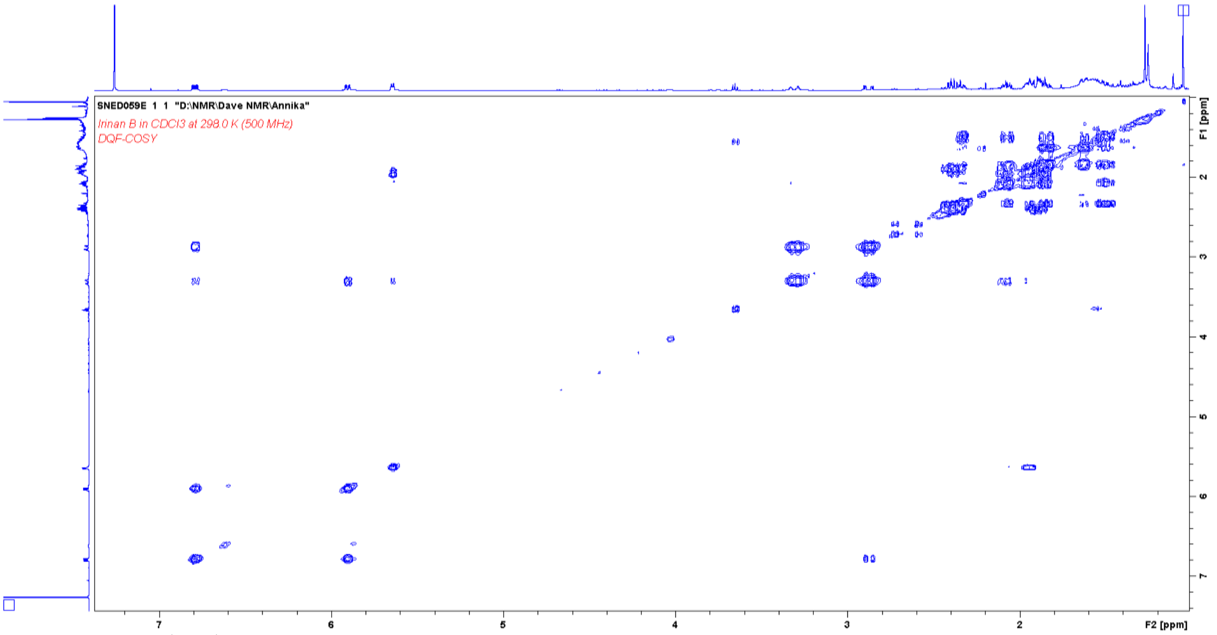

Figure S13. ^{1}H-^{1}H COSY spectrum of **3** in $CDCl_3$ at 298.0 K (500 MHz).

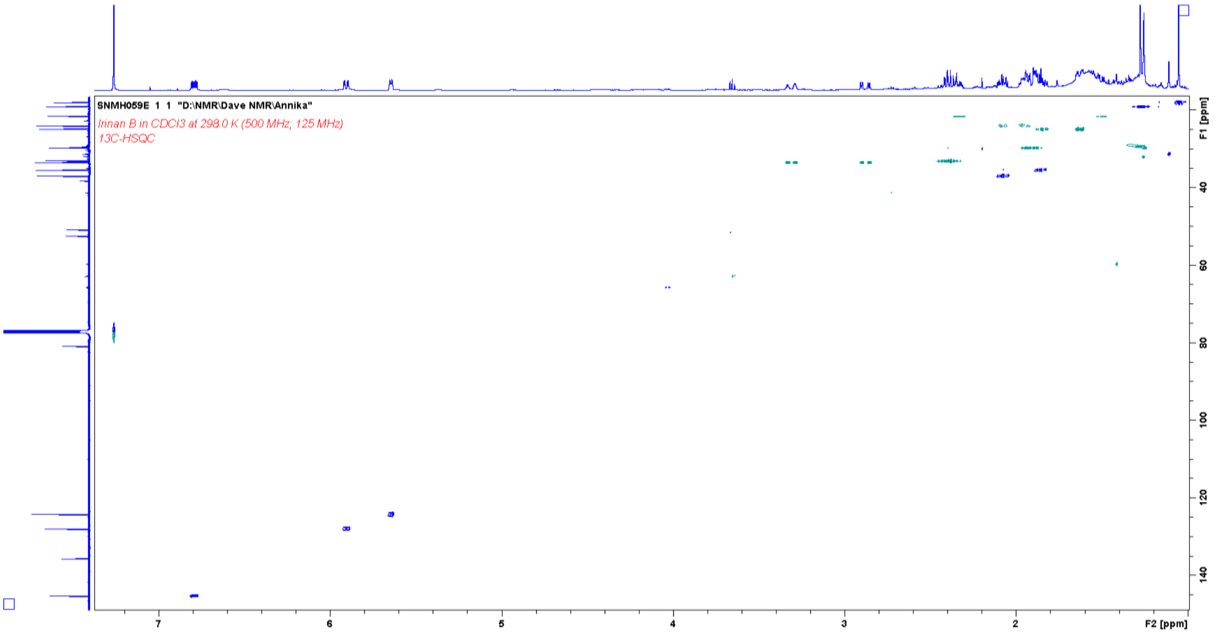

Figure S14. HSQC spectrum of **3** in $CDCl_3$ at 298.0 K (500 MHz; 125 MHz).

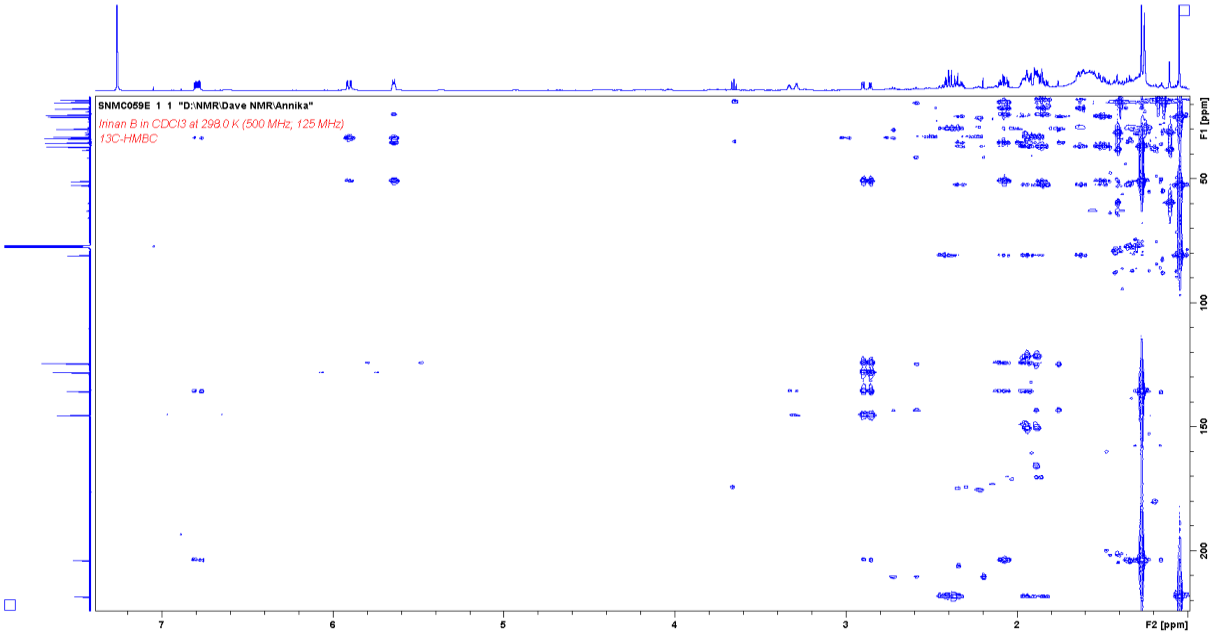

Figure S15. HMBC spectrum of **3** in $CDCl_3$ at 298.0 K (500 MHz; 125 MHz).

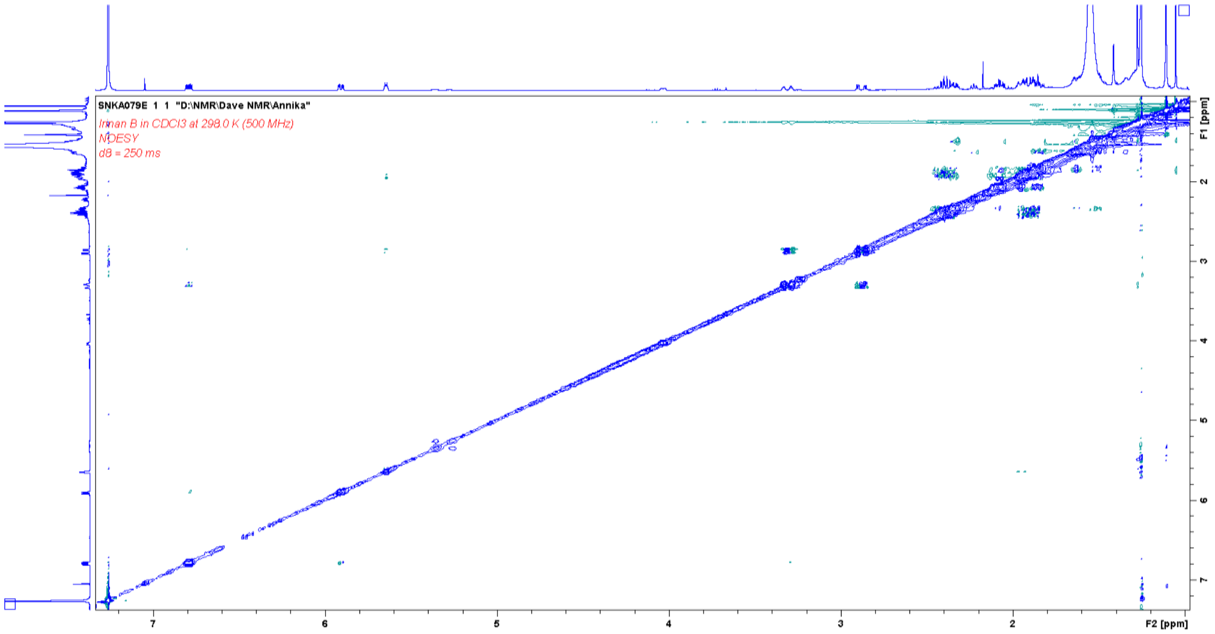

Figure S16. NOESY spectrum of **3** in $CDCl_3$ at 298.0 K (500 MHz).

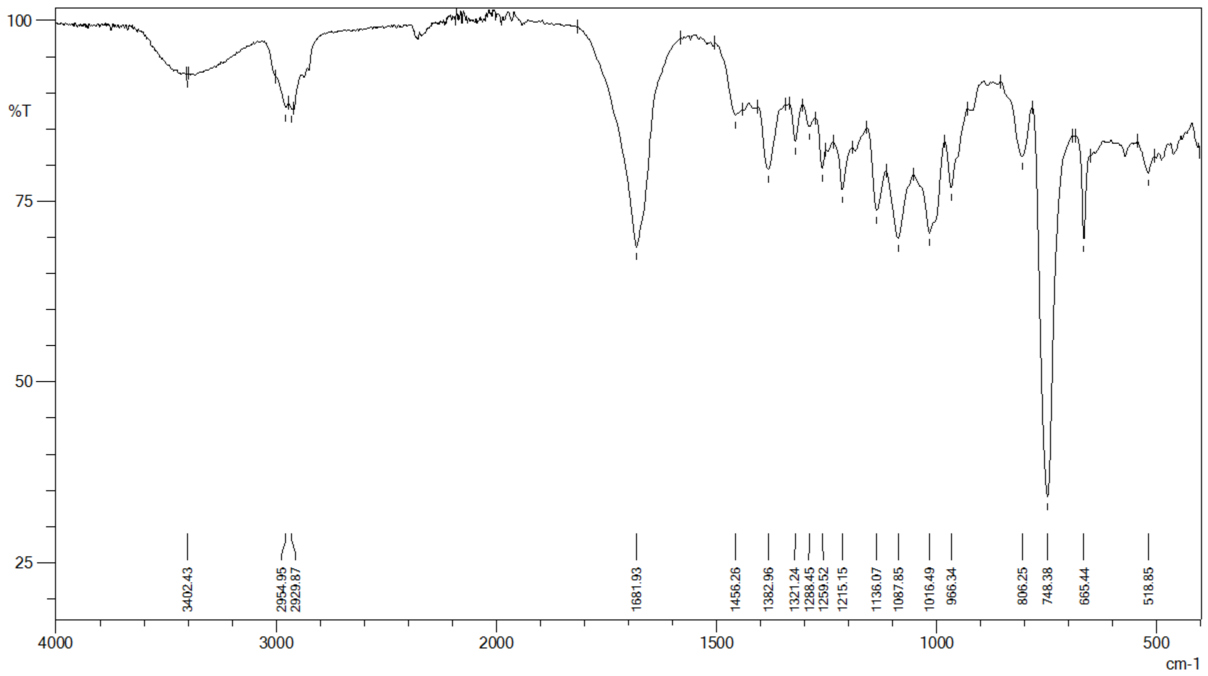

Figure S17. IR spectrum of **3**.

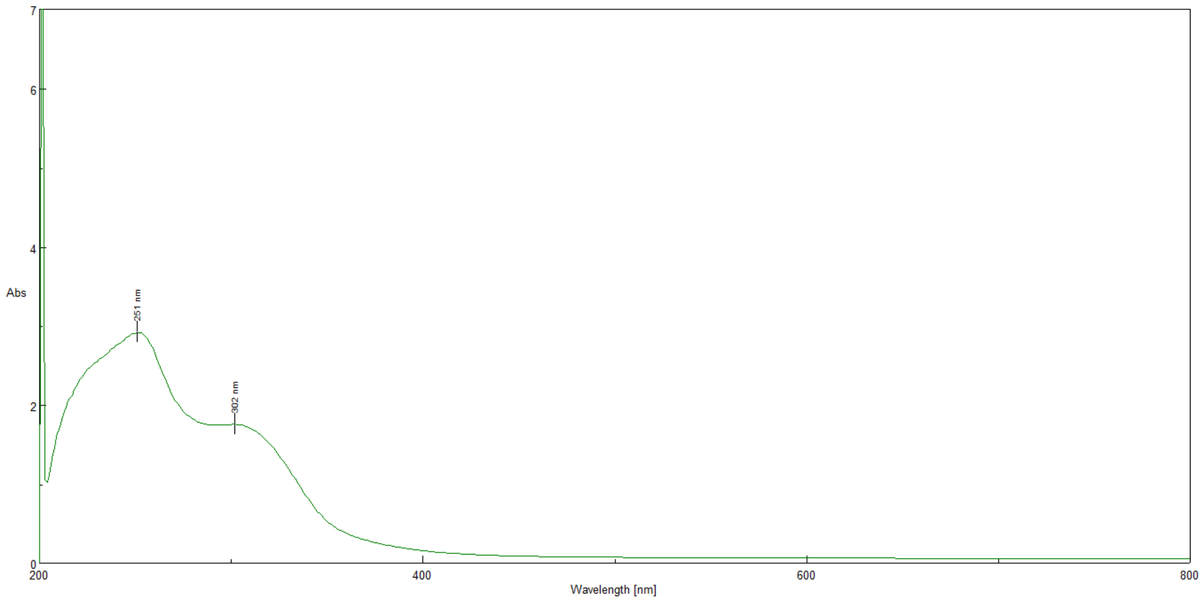

Figure S18. UV–vis spectrum of **3** in MeOH.

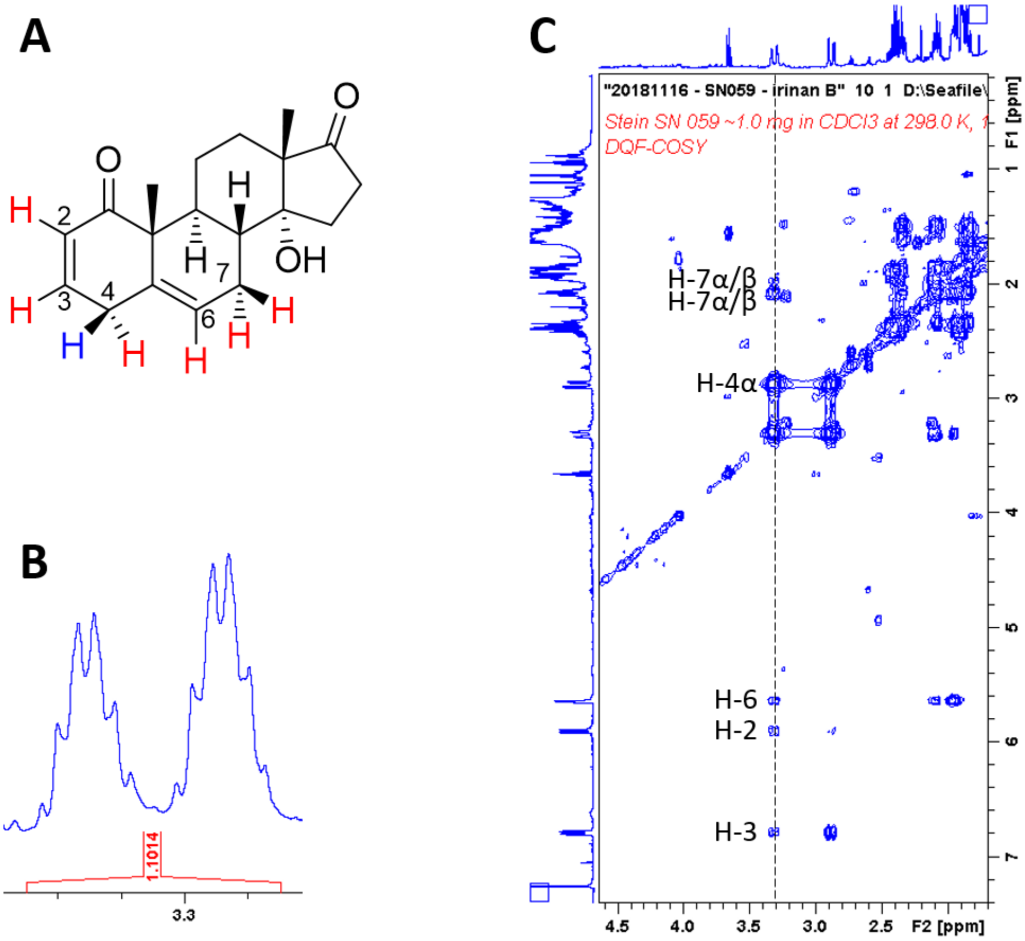

Figure S19. Origin of an unusual dddddd multiplet. A) Coupling partners (red) of H-4β (blue) in irinan B (**3**). B) H-4β appears as a dsext (expected for a sextet 1:5:10:10:5:1) in a ^{1}H NMR spectrum (500 MHz, $CDCl_3$, 298 K). C) COSY analysis reveals ^{2}J coupling to H-4α (21.3 Hz) and five additional correlations (which must be ≈2.8 Hz to explain the sextet).

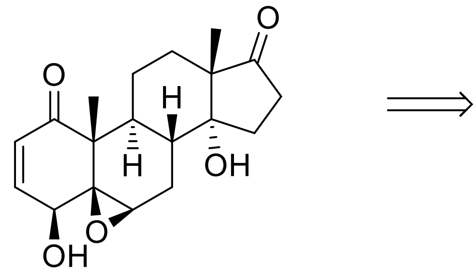

Irinan A (**2**)
Physalis peruviana

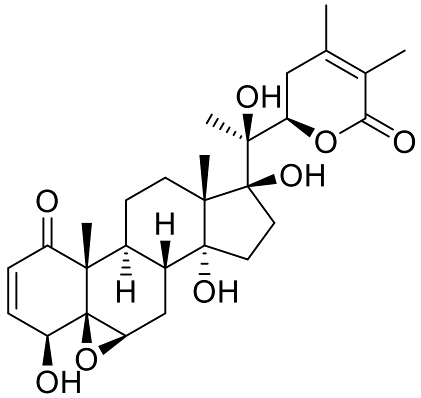

4ß-Hydroxywithanolide E (**1**)

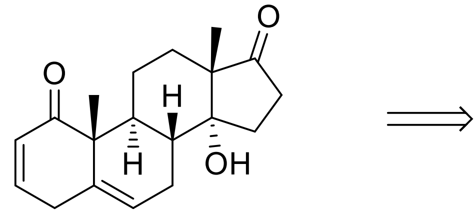

Irinan B (**3**)
Physalis peruviana

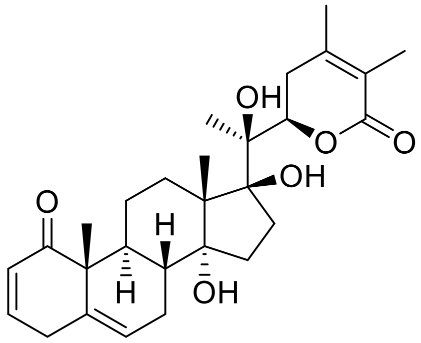

Withanolide F (**5**)

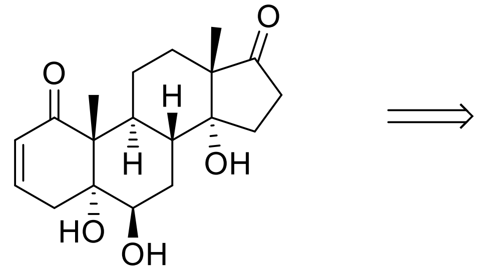

Cinedione (**8**)
Physalis cinerascens

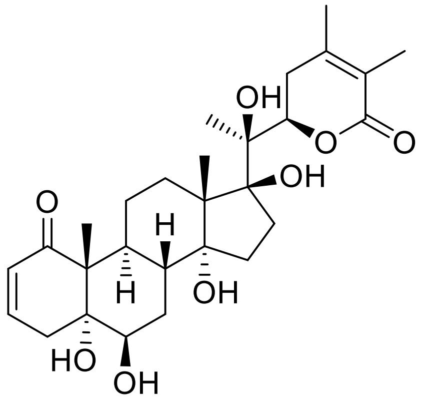

Withanolide S

Figure S20. Proposed biosynthetic precursors of irinan A (**2**), B (**3**) and the previously reported androwithanolide cinedione (**8**).

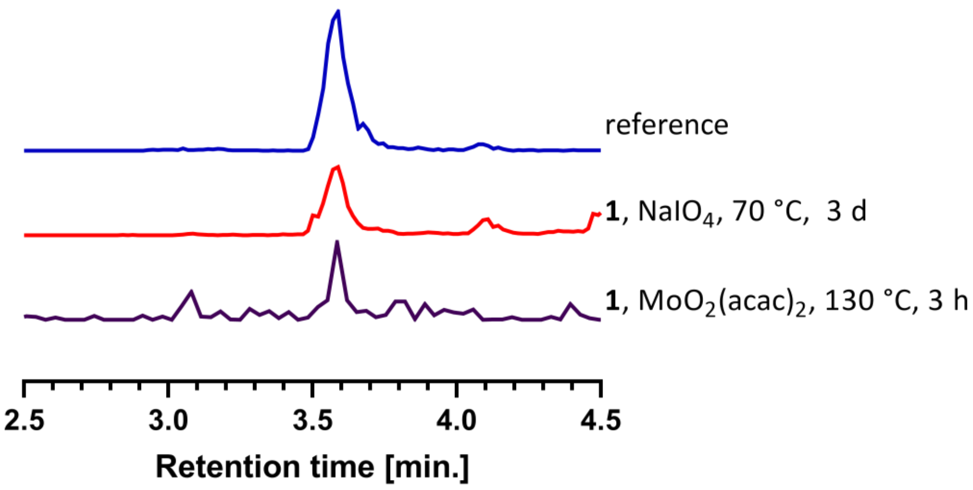

Figure S21. Intrinsic reactivity of 4ß-hydroxywithanolide E (**1**) in oxidative conditions. Formation of irinan A (**2**) as judged by extracted ion chromatograms of *m*/*z* 315 $[M + H - H_2O]^+$ in comparison to an authentic irinan A (**2**) reference.

Table S1. ^{1}H NMR data ($CDCl_3$, 400 MHz, 298 K) of withanolide E (**4**), withanolide F (**5**) and perulactone H (**6**) in comparison with literature data (δ in ppm, *J* in Hz).

	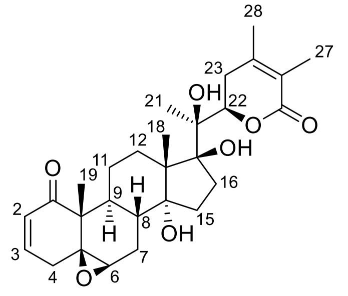 Withanolide E (**4**)		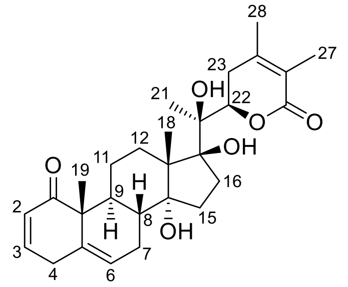 Withanolide F (**5**)		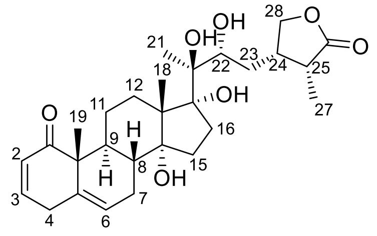 Perulactone H (**6**)	
Atom	^{1}H	reference [1]	^{1}H	reference [1]	^{1}H	reference [1]
H-2	6.03 dd (10.1, 2.9)	6.02 dd (10.1, 2.8)	5.86 dd (10.0, 2.0)	5.86 br dd (9.9, 1.8)	5.91 dd (9.9, 2.1)	5.90 dd (9.9, 1.7)
H-3	6.81 ddd (10.2, 6.4, 2.4)	6.81 ddd (10.1, 6.4, 2.8)	6.76 ddd (10.0, 5.0, 2.5)	6.76 ddd (9.9, 4.6, 2.3)	6.79 ddd (10.1, 4.8, 2.5)	6.79 ddd (9.9, 4.9, 2.3)
H-4	n.d. [a]	not reported	3. 27 br dd (21.4, 1.7)<br>2.83 dd (21.1, 5.0)	3.27 br dd (21.1, 2.3)<br>2.83 dd (21.1, 4.6)	3.30 br d (21.0)<br>2.86 dd (21.2, 4.3)	3.30 br dd (21.2, 2.3)<br>2.87 dd (21.2, 4.9)
H-6	3.18 br s	3.18 br s	5.61 m	5.60 m	5.61 m	5.61 m
H-7	n.d. [a]	not reported	n.d. [a]	not reported	n.d. [a]	1.86-1.95 m
H-8	n.d. [a]	not reported	n.d. [a]	not reported	n.d. [a]	1.92 m
H-9	n.d. [a]	not reported	n.d. [a]	not reported	n.d. [a]	2.13 m
H-11	n.d. [a]	not reported	n.d. [a]	not reported	n.d. [a]	2.36 m, 1.65 m
H-12	n.d. [a]	not reported	n.d. [a]	not reported	n.d. [a]	2.34 m, 1.58 m
H-15	n.d. [a]	not reported	n.d. [a]	not reported	n.d. [a]	1.77 m, 1.62 m
H-16	n.d. [a]	not reported	n.d. [a]	not reported	n.d. [a]	2.63 ddd (15.2, 10.9, 1.7)<br>1.88 m
H-18	1.09 s	1.09 s	1.13 s	1.13 s	0.99 s	0.99 s
H-19	1.24 s	1.24 s	1.24 s	1.23 s	1.27 s	1.27 s
H-21	1.41 s	1.41 s	1.43 s	1.42 s	1.27 s	1.27 s
H-22	4.89 dd (11.4, 4.8)	4.89 dd (11.5, 5.1)	4.95 dd (9.9, 6.6)	4.94 dd (9.4, 6.4)	3.74 dd (10.5, 2.1)	3.74 d (10.3)
H-23	2.51 m	2.51 m	n.d. [a]	not reported	n.d. [a]	1.79 m, 1.62 m
H-24	-	-	-	-	n.d. [a]	2.78 m
H-25	-	-	-	-	n.d. [a]	2.68 m
H-27	1.88 s	1.88 s	1.89 s	1.89 s	1.20 d (7.6)	1.20 d (7.5)
H-28	1.94 s	1.94 s	1.95 s	1.94 s	4.48 dd (9.2, 7.2)<br>4.11 dd (9.0, 8.4)	4.48 dd (9.2, 7.2)<br>4.11 dd (9.2, 9.2)

[a] not determined using ^{1}H NMR data

Supporting references

1. Ozawa, M.; Morita, M.; Hirai, G.; Tamura, S.; Kawai, M.; Tsuchiya, A.; Oonuma, K.; Maruoka, K.; Sodeoka, M. *ACS Med. Chem. Lett.* **2013**, *4*, 730–735.
